# Commonalities and differences in ECT-induced gray matter volume change between depression and schizophrenia

**DOI:** 10.1016/j.nicl.2023.103429

**Published:** 2023-05-03

**Authors:** Hirotsugu Kawashima, Shimpei Yamasaki, Manabu Kubota, Masaaki Hazama, Yasutaka Fushimi, Jun Miyata, Toshiya Murai, Taro Suwa

**Affiliations:** a Department of Psychiatry, Graduate School of Medicine, Kyoto University, Kyoto, Japan; b Department of Diagnostic Imaging and Nuclear Medicine, Graduate School of Medicine, Kyoto University, Kyoto, Japan

**Keywords:** Electroconvulsive therapy, Magnetic resonance imaging, Voxel-based morphometry, Major depressive disorder, Schizophrenia

## Abstract

**Background:**

Electroconvulsive therapy (ECT) is one of the most effective treatments for depression and schizophrenia, particularly in urgent or treatment-resistant cases. After ECT, regional gray matter volume (GMV) increases have been repeatedly reported both in depression and schizophrenia. However, the interpretation of these findings remains entangled because GMV changes do not necessarily correlate with treatment effects and may be influenced by the intervention itself. We hypothesized that the comparison of longitudinal magnetic resonance imaging data between the two diagnostic groups will provide clues to distinguish diagnosis-specific and transdiagnostic changes.

**Method:**

Twenty-nine Japanese participants, including 18 inpatients with major depressive disorder and 11 with schizophrenia, underwent longitudinal voxel-based morphometry before and after ECT. We investigated GMV changes common to both diagnostic groups and those specific to each group. Moreover, we also evaluated potential associations between GMV changes and clinical improvement for each group.

**Results:**

In both diagnostic groups, GMV increased in widespread areas after ECT, sharing common regions including: anterior temporal cortex; medial frontal and anterior cingulate cortex; insula; and caudate nucleus. In addition, we found a schizophrenia-specific GMV increase in a region including the left pregenual anterior cingulate cortex, with volume increase significantly correlating with clinical improvement.

**Conclusions:**

Transdiagnostic volume changes may represent the effects of the intervention itself and pathophysiological changes common to both groups. Conversely, diagnosis-specific volume changes are associated with treatment effects and may represent pathophysiology-specific impacts of ECT.

## Highlights

• Volume change after ECT can be either diagnosis-specific or transdiagnostic.
• Comparing two diagnostic groups may provide clues to distinguish them.
• Common increase in regions including the limbic system may be trandiagnostic.
• Pregenual anterior cingulate cortex shows schizophrenia-specific volume increase.
• Volume increase in this region correlates with clinical improvement.

## 1 Introduction

Electroconvulsive therapy (ECT) is one of the most effective treatments for depression. (UK Ect Review Group, 2003, Lisanby, 2007) It also plays a vital role in schizophrenia (SCZ) treatment, especially when rapid improvement of symptoms is required, such as catatonia. In addition, ECT is one of the therapeutic options for treatment-resistant schizophrenia. (Grover et al., 2019, Pompili et al., 2013) However, its mechanism of action remains unclear despite various attempts both in animal and clinical studies. Recent longitudinal magnetic resonance imaging (MRI) studies (Oltedal et al., 2018, Sartorius et al., 2019, Joshi et al., 2016, Takamiya et al., 2019) have demonstrated that ECT increases gray matter volume (GMV) in patients with depression, especially in the hippocampus and amygdala, for which neuroplastic mechanisms including neurogenesis have been postulated. However, the results on whether brain volume increases are associated with therapeutic effects are inconsistent, and previous *meta*-analyses have found no association. (Gbyl and Videbech, 2018, Takamiya et al., 2018, Gryglewski et al., 2021) In a recent multicenter study with a large sample size, widespread brain regions, not restricted to the hippocampus and amygdala, showed volume increases. (Ousdal et al., 2020) These volume increases correlated with the strength of the stimulating electric field but not with the therapeutic effect. (Argyelan et al.,) Similar longitudinal imaging studies have been conducted in SCZ, (Wolf et al., 2016, Thomann et al., 2017, Jiang et al., 2019, Jiang et al., 2019, Wang et al., 2019, Shan et al., 2021) although fewer in number than in depression. These studies demonstrated volume increase in the hippocampus, amygdala, insula, and other regions. However, the insight into whether these volume increases are associated with therapeutic effects remains inconclusive. (Moon et al., 2021).

Multiple factors hinder the interpretation of these volumetric effects of ECT. For example, these volume changes can encompass the effects related to the diagnosis and those related to the intervention (e.g., changes associated with electric stimulus and induced seizures). These possible causative factors are difficult to distinguish from each other when investigated in a single diagnostic group or when examined by comparing diagnostic and healthy control groups. In this study, we considered comparing changes after ECT between two diagnostic groups (depression and schizophrenia) to address this issue. Volume changes common to both diagnostic groups may be interpreted as transdiagnostic changes. Conversely, volume changes specific to a single diagnostic category might be interpreted as pathophysiology-specific impacts of ECT.

Hence, we performed voxel-based morphometry (VBM) before and after ECT in patients with depression and those with SCZ with the same image processing and analyzing protocol. We aimed to examine regional GMV changes common to both diagnostic groups and those specific to each diagnostic group. Furthermore, we investigated the association between GMV changes and clinical improvement after ECT for each group.

## 2 Method

### 2.1 Participants

Twenty-nine Japanese participants were enrolled, including 18 inpatients with major depressive disorder (MDD) and 11 inpatients with SCZ. They were recruited from the Department of Psychiatry at Kyoto University Hospital in Kyoto, Japan. All patients met all criteria for MDD or SCZ according to the Diagnostic and Statistical Manual of Mental Disorders, Fourth Edition, Text Revision or Fifth Edition (DSM-IV-TR or −5). None of the participants had a history of severe medical conditions; current substance abuse; or a history of seizure disorder, cerebrovascular disease, or brain injury. The authors assert that all procedures contributing to this work comply with the ethical standards of the relevant national and institutional committees on human experimentation and with the Helsinki Declaration of 1975, as revised in 2008. All procedures involving patients were approved by the Committee on Medical Ethics of Kyoto University (C0810-12). Written informed consent was obtained from all participants, except those who could not give consent because of catatonia, for whom written informed consent was acquired from their proxies.

### 2.2 ECT procedure

All patients met the application criteria for ECT according to the American Psychological Association task force guidelines. Bitemporal ECT was administered twice a week using a brief-pulse square-wave ECT device (Thymatron System IV; Somatics, LLC, Venice, FL, USA). Before ECT, benzodiazepines and mood stabilizers (i.e., lithium, valproate, carbamazepine, and lamotrigine) were discontinued, while antidepressants and antipsychotics were continued. Anesthesia and muscle relaxation were intravenously induced using propofol (1–2 mg/kg body weight) and succinylcholine chloride (1 mg/kg), respectively. The stimulus intensity for the first ECT session was determined according to the half-age method, and the subsequent intensity was adjusted according to seizure quality (monitored with motor seizure and electroencephalogram manifestations). ECT sessions were performed until the patient reached a plateau of improvement over the two previous sessions.

### 2.3 Clinical assessment

The MDD and SCZ groups were evaluated with the Hamilton 17-item Depression Rating Scale (HDRS) and Brief Psychiatric Rating Scale (BPRS), respectively, before and after the acute ECT course.

### 2.4 MRI acquisition

Using a 3-Tesla MRI scanner with a 32-channel head coil (MAGNETOM Skyra®, Siemens Healthineers, Erlangen, Germany), we scanned each patient twice (within a week prior to the first ECT session and within a week after the final ECT session). We acquired high-resolution anatomical images in the sagittal mode by using three-dimensional (3D) T1-weighted magnetization-prepared rapid acquisition of a gradient echo (MPRAGE; repetition time = 2300 ms, echo time = 2.98 ms, field of view = 232 × 256 mm, flip angle = 9°, 224 slices, matrix = 232 × 256, and final voxel size = 1 × 1 × 1 mm).

### 2.5 Image processing

Preprocessing was performed using the Computational Anatomy Toolbox (CAT12.8; https://www.neuro.uni-jena.de/cat) implemented in the Statistical Parametric Mapping software package (SPM12; The Wellcome Department of Imaging Neuroscience, London, UK). The acquired MPRAGE data were visually checked for gross anomalies and artifacts and reoriented to adjust image origins at the anterior commissure. We used a longitudinal preprocessing pipeline for detecting small changes in CAT12. After an initial intrasubject rigid registration, which includes bias correction between time points, the realigned images of each time point were segmented into gray matter, white matter, and cerebrospinal fluid. Subsequently, the registration parameters (i.e., deformation fields) were estimated using Geodesic Shooting and then averaged. Next, the resulting mean deformation was applied to the tissue segmentations of each time point and modulated with the Jacobian determinant of the deformation field. Finally, the modulated gray matter images were smoothed using a Gaussian kernel of 8 mm full-width at half-maximum.

### 2.6 Statistical analysis

#### 2.6.1 Demographic data and clinical characteristics

Demographic and clinical data were analyzed using SPSS statistics version 27 (IBM, Armonk, NY, USA).

#### 2.6.2 Comparison of the regions showing GMV changes after ECT between the MDD and SCZ groups

Two-way repeated measures analysis of covariance (ANCOVA) was conducted for each voxel of gray matter in SPM12, with diagnosis (MDD or SCZ) as the between-subjects factor and time (pre- or post-ECT) as the within-subject factor. Covariates included age, sex, total number of ECT sessions, and total intracranial volume (Supplementary Fig. 1). In this analysis, the group-by-time interaction effect represents regions where GMV changes differ over time between the two groups, whereas the simple main effect of time shows areas with longitudinal GMV changes within each group.

**Fig. 1:**
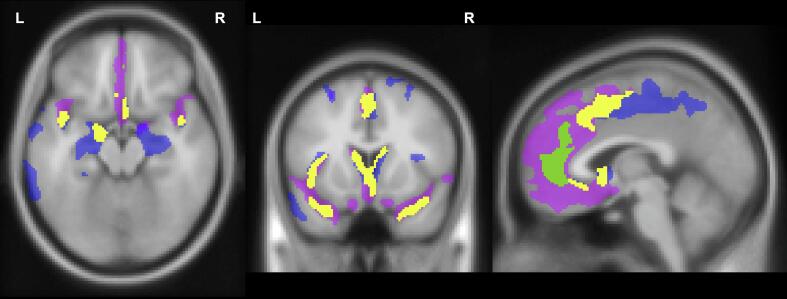
Gray matter volume increases after ECT. The results are overlaid: the regions indicated by the simple main effects of time in the MDD (blue) and SCZ (pink) groups; the areas showing increases common to both groups demonstrated with the conjunction analysis (yellow); the region showing a significant interaction of group by time (green). Results are displayed with a significance threshold of *p* < 0.05, with family-wise error correction at the cluster level. MDD, major depressive disorder; SCZ: schizophrenia.

To detect commonalities in volume increases between the two groups, we performed a conjunction analysis for testing the conjunction null hypothesis. The logical conjunction for this purpose here was defined as follows: (pre-ECT MDD < post-ECT MDD) ∩ (pre-ECT SCZ < post-ECT SCZ) (Supplementary Fig. 2).

**Fig. 2:**
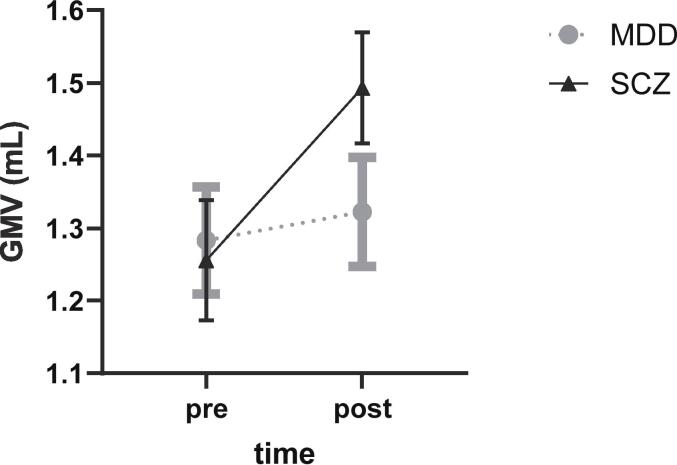
Schizophrenia-specific GMV increase. GMV increase after ECT was more prominent in the SCZ group than that in the MDD group in the cluster showing a significant group-by-time interaction effect in the two-way repeated measured analysis of covariance. Error bars represent standard error of the mean. GMV, gray matter volume; SCZ, schizophrenia; MDD, major depressive disorder.

For these SPM analysis described above, we applied uncorrected *p* < 0.001 for the cluster-forming threshold and *p* < 0.05 for the cluster-wise family-wise error (FWE) threshold. According to the automated anatomical labeling 3 (AAL3) atlas implemented in xjView 10.0 (https://www.alivelearn.net/xjview), we determined the major brain regions included in the detected clusters.

For clusters that showed a significant group-by-time interaction, GMVs within the region at before and after ECT in each group were calculated; subsequently, post-hoc comparisons between groups were performed using SPSS.

#### 2.6.3 Association between regional GMV change and clinical symptom improvement

We explored the association between symptom improvement and GMV change for each group. First, symmetrized percent change (SPC) images between pre- and post-ECT were calculated [SPC = 100 × (post-ECT – pre-ECT) / (post-ECT + pre-ECT)] using the ImCalc function of SPM12. Subsequently, we conducted multiple regression analyses, using the SPC images as dependent variables and the percent change of clinical symptom scales (HDRS and BPRS for the MDD and SCZ groups, respectively) as the independent variables. Age, sex, and the total number of ECT sessions were included as confounding covariates. We applied uncorrected *p* < 0.001 for the cluster-forming threshold and *p* < 0.05 for the cluster-wise FWE threshold.

For a region that showed a significant group-by-time interaction, the most prominent anatomical location within the region was identified on the AAL3 atlas and considered a region of interest. After confirming normality with Shapiro–Wilk test, the correlation between volume changes and percent changes of clinical symptom improvement was examined using Pearson’s correlation coefficient. This correlation analysis was performed in SPSS.

## 3 Results

### 3.1 Demographic data and clinical characteristics

Table 1 lists the participants’ demographics and clinical characteristics. The mean age of the SCZ group was significantly lower than that of the MDD group. Catatonia was present in nine patients in the SCZ group and one in the MDD group. The remaining two patients in the SCZ group underwent ECT because of treatment resistance. ECT was generally effective in decreasing clinical rating scale scores. Out of 18 patients with MDD, 13 matched the clinical response criteria (defined as > 50% decrease in HDRS score), of which 7 patients fulfilled the criteria for remission (HDRS < 8). In the SCZ group, 9 out of 11 patients showed clinical response (defined as > 50% decrease in BPRS score), whereas 2 demonstrated partial response (defined as > 25% improvement).

**Table 1:** Demographic and clinical characteristics.

Demographic and clinical variables		MDD patients (n = 18)		SCZ patients (n = 11)		P-value
Mean age, years (SD)		61.9	(16.0)	44.9	(7.9)	0.004^†^
Sex, female (*n* [%])		9	(50.0)	5	(45.5)	0.812^‡^
Psychotic Depression (*n* [%])		9	(50.0)			
Catatonia (*n* [%])		1	(5.6)	9	(81.8)	
Mean number of ECT sessions (SD)		9.6	(2.8)	9.1	(2.9)	0.645^§^
Clinical Score						
mean HDRS (SD)	pre ECT	27.6	(7.3)			<0.001^¶^
	post ECT	9.7	(6.0)			
mean BPRS (SD)	pre ECT			57.3	(9.8)	<0.001^¶^
	post ECT			18.3	(9.2)	

MDD: major depressive disorder, SCZ: schizophrenia, SD: standard deviation, HDRS: Hamilton Depression Rating Scale, BPRS: Brief Psychotic Rating Scale.

†: Mann–Whitney-*U* test, ‡: Chi-squared test, §: two-sample *t*-test, ¶: paired *t*-test,

### 3.2 Comparison of the regions showing GMV changes after ECT between the MDD and SCZ groups

The simple main effect of time in the MDD group revealed 13 significant clusters of GMV increase, including bilateral regions spreading from the hippocampus to the medial temporal lobe and insula, as well as the medial cerebral hemisphere from the middle cingulate cortex to the precuneus (see the blue part of Fig. 1). Detailed information is shown in Supplementary Table S1. In the SCZ group, the simple main effect of time showed four significant clusters of GMV increase in the bilateral medial temporal lobe and insula, bilateral medial frontal lobe, and left superior and middle frontal gyrus (see the pink part of Fig. 1). Detailed information is shown in Supplementary Table S2. Each group had no suprathreshold clusters showing a GMV decrease.

These clusters of GMV increase in both groups were broadly distributed with some overlaps. Conjunction analysis showed four clusters of GMV increase, including the following regions: (1) left temporal pole/superior temporal gyrus/insula/amygdala/hippocampus/rolandic operculum, (2) bilateral caudate/olfactory cortex/subgenual anterior cingulate gyrus, (3) bilateral middle cingulate gyrus/supplementary motor area/medial superior frontal gyrus, (4) right temporal pole/superior temporal gyrus/parahippocampal gyrus (see the yellow part of Fig. 1). Supplementary Table S3 presents more detailed information.

The group-by-time interaction effect was significant in a cluster including the bilateral pregenual anterior cingulate cortex (pACC) (see the green part of Fig. 1 and Table 2). For GMV within this cluster, the post-hoc two-way repeated measures analysis of variance revealed a significant difference in the interaction effects of group-by-time [F(1,27) = 22.77, p < 0.0001] (Fig. 2). Although GMV increases were significant in each group according to paired t-tests, they were more prominent in the SCZ group (Cohen’s d = 1.611) than in the MDD group (Cohen’s d = 0.516) (Supplementary Fig. 3).

**Table 2:** Brain regions showing significant group-by-time interaction effect on grey matter volume in MDD and SCZ patients.

Brain regions	Cluster				MNI coordinate			Peak T value	AAL3	
	Size	p (FWE-corr)	p (FDR-corr)		X	Y	Z		# of voxels	Structure
Bilateral pACC/medial Frontal	1058	0.0005	0.0005		−1.5	45	4.5	5.28	576	ACC_pre_L
					−1.5	37.5	34.5	3.95	152	Frontal_Sup_Medial_L
					−1.5	37.5	25.5	3.80	123	ACC_pre_R

MNI: Montreal Neurological Institute; pACC: pregenual anterior cingulate cortex; FWE-corr: Family-wise Error correction; FDR-corr: False Discovery Rate correction; AAL3: Automated Anatomical Labelling atlas 3; # of voxels: total number of voxels; ACC_pre_L: left pregenual anterior cingulate cortex; Frontal_Sup_Medial_L: left superior frontal gyrus medial part; ACC_pre_R: right pregenual anterior cingulate cortex.

**Fig. 3:**
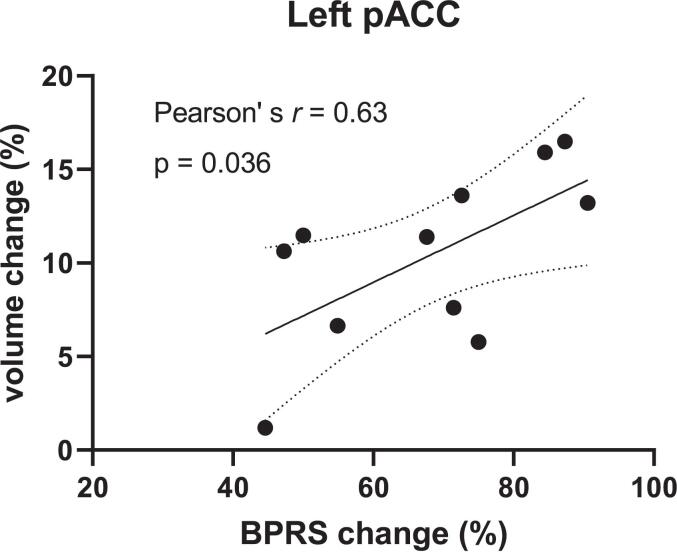
Correlation between symptom improvement and volume increase in the left pACC region. GMV changes in the left pACC region in the SCZ group was significantly correlated with the percentage changes in BPRS. pACC, pregenual anterior cingulate cortex; GMV, gray matter volume; BPRS, Brief Psychiatric Rating Scale.

Because of the significant age differences between the two groups, we performed the same analysis for this region in a subset of the patients with MDD whose ages were matched with those of patients with SCZ. Of 18 participants in the MDD group, 11 were selected in ascending order of age, matching the number of participants in the SCZ group. No significant differences were observed in the mean age between the two groups (p = 0.173, Supplementary Table S4). As a result, significant group-by-time interaction effect [F(1,20) = 13.03, p = 0.0017] (Supplementary Fig. 4) and GMV increases in each group were replicated (Supplementary Fig. 5). Thus, the differences in GMV increases within this region was unlikely to be attributed to age.

### 3.3 Association between regional GMV change and clinical symptom improvement

We found no suprathreshold cluster that showed a positive or negative correlation with the reduction rates of the clinical symptom scale. As demonstrated above, a region showed a significant group-by-time interaction effect. According to the AAL3 atlas, this region was mainly the left pACC, showing a GMV increase specific to the SCZ group. In the SCZ group, GMV changes in the left pACC region were found to be significantly correlated with percent changes on BPRS (Fig. 3).

## 4 Discussion

To the best of our knowledge, this is the first VBM study to examine and compare GMV changes after bilateral ECT between patients with MDD and those with SCZ. While the volume increases in each group overlapped in several regions, including the hippocampus, amygdala, and insula, the extent of the volume increases differed between the MDD and SCZ groups. In particular, the area including the bilateral pACC showed a significant volume increase, specifically in the SCZ group. The results of the present study indicate the possibility of distinguishing transdiagnostic GMV changes from pathophysiology-specific GMV changes associated with therapeutic efficacy.

The regions of GMV volume increase in the MDD group are generally consistent with previous studies (Abbott et al., 2014;4:e483-., Ota et al., 2015, Bouckaert et al., 2016, Jorgensen et al., 2016, Pirnia et al., 2016, Cano et al., 2017) that demonstrated significant GMV increases after ECT for patients with depression. In the present study, the GMV percent change was not associated with the HDRS percent change; however, our previous study, (Yamasaki et al., 2020) which included 14 patients with MDD, reported that the GMV increase in the left hippocampus was associated with an improvement in depressive symptoms. Results from previous studies investigating the association between GMV changes and symptom improvement have also been inconsistent. Thus, this issue remains controversial and requires further investigation. Regarding the SCZ group, previous studies that examined volume changes after ECT reported volume increases mainly in the medial temporal lobe, (Takamiya et al., 2018, Gryglewski et al., 2021) including the hippocampus/parahippocampal gyrus and amygdala, (Ousdal et al., 2020) and in the insula. (Wolf et al., 2016, Thomann et al., 2017) Moreover, some studies reported volume increases in the right temporal pole and superior temporal gyrus (Wolf et al., 2016) and the left superior temporal gyrus and middle temporal gyrus. (Thomann et al., 2017) The present results concur with these findings. However, our results also showed volume increases in the medial frontal lobe, including the pACC and the supplementary motor cortex. Significant volume increases in these regions have not previously been reported in SCZ.

In the present study, a significant group-by-time interaction effect for GMV increase was observed in the region including the pACC. Furthermore, GMV changes of this region significantly correlated with the BPRS percent change. Thus, the GMV increase in this region was a change associated with the therapeutic effect specific to patients with SCZ who had undergone ECT. In many studies, ACC volume consistently decreased in patients with various stages of SCZ, with lower variability. (Brugger and Howes, 2017) Therefore, abnormalities in the ACC region might be a potential core of the pathophysiological basis of SCZ. (Ochi et al., 2022) Moreover, pACC, which has extensive connections with the amygdala, (Devinsky et al., 1995) is associated with emotional function. Additionally, pACC is structurally and functionally distinct from other ACC regions but remains poorly understood compared with other regions. (Palomero-Gallagher et al., 2019) Further research on pACC function and SCZ pathophysiology is needed.

The regions showing GMV increase after ECT largely overlapped in both groups, supporting a common mechanism of brain plasticity with ECT irrespective of the pathophysiological conditions to which the intervention is administered. Thomann et al. (Thomann et al., 2017) reported ipsilateral volume increase in the medial temporal lobe comprising the hippocampus, amygdala, and insula in patients from both MDD and SCZ groups who underwent right unilateral ECT. In our study, similar volume increases in both hemispheres after ECT may be attributed to the bilateral electrode placement; therefore, common GMV changes in these regions may be associated with the intervention. We also found that the areas of volume increase common to both groups showed a broader extent than those reported in Thomann et al.’s study. This difference may be explained by the fact that our study had a slightly larger sample size than Thomann et al.’s study, (Thomann et al., 2017) where 12 individuals with MDD and 9 with SCZ were included.

Regarding the strengths of this study, we used conjunction analysis to detect common regions of volume increase. Thomann et al. examined the main effect of time to reveal the common regions of GMV increases using two-way repeated measures ANCOVA. However, their results cannot necessarily be referred to as common regions of volume increase for both groups because their analysis detected regions of volume increase collectively for both groups. In contrast, conjunction analysis is a relatively conservative approach because it strictly limits the occurrence of false positive voxels to reject the conjunction null hypothesis. (Friston et al., 2005) Therefore, compared to the method used by Thomann et al., it provides more robust results for commonality. Moreover, the study by Thomann et al. did not report interaction effects; therefore, they could not confirm which regions of volume increase differed between the depression and SCZ groups. The present study addressed these issues, thereby providing more solid findings regarding the commonalities and differences in changes after ECT between the two diagnostic groups.

### 4.1 Limitation

This study has several limitations. First, the sample size was small and imbalanced because of its exploratory nature. Thus, the SCZ group with a smaller sample size might be more likely to have voxels that were not reflected as significant differences, even when the effect size was the same as that of the MDD group. A larger and well-balanced sample size is needed in future studies to generalize our findings. Second, the significant difference in the mean age between the two groups may lead to bias. Since GMV changes after ECT might be correlated with age, (Takamiya et al., 2018, Deng et al., 2015) the difference in volume changes could be driven by age. To address this, we performed age-matched comparisons and replicated similar results. Although we considered the differences in age have little impact on the results in the regions examined in this study, it is desirable to ensure that the participants’ backgrounds are comparable in future studies. Thirdly, we could not exclude the possible confounding effects of concurrent pharmacotherapy comprising various kinds of psychotropic agents, such as antipsychotics, antidepressants, and mood stabilizers. Fourth, most of our patients with SCZ exhibited catatonia. The pathogenesis of catatonia includes supplementary motor area and medial frontal lobe dysfunction.(Walther et al., 2019) Thus, the increased volume in these areas, as observed in the present study’s results, might be caused by catatonia rather than SCZ itself. Therefore, caution should be exercised when generalizing our results to patients with SCZ as a whole. Finally, it is methodologically difficult to distinguish the transdiagnostic changes associated with the intervention itself and the common pathophysiological changes shared by MDD and SCZ. Comparing these groups with another diagnostic group with a larger sample size might allow these factors to be distinguished more thoroughly.

## 5 Conclusion

This study identified the commonalities and differences in GMV increases after ECT in the MDD and SCZ groups, indicating that some parts in the mechanism of ECT are common between diagnoses, whereas some parts differ in each diagnosis. In particular, the region that primarily comprised pACC exhibited a volume increase specific to the SCZ group. This volume increase correlated with symptom improvement. Further research on the role of this region in SCZ (or catatonia) is warranted. Thus, comparing post-ECT changes between the two diagnostic groups would provide some clues to differentiate diagnosis-specific changes from transdiagnostic ones. This approach has not yet been extensively used, and replicating it with similar methods in a larger sample size is desirable.

## 6 Data availability

Data supporting the findings of this study are available from the corresponding author (H.K.) upon reasonable request.

## Funding

This work was supported by Japan Society for the Promotion of Science (JSPS) KAKENHI (Grant Numbers JP21H02849, JP26461743, and JP21H05173) and the Strategic International Brain Science Research Promotion Program (Brain/MINDS Beyond) from Japan Agency for Medical Research and Development (AMED) (Grant Numbers JP22dm0307102, JP22dm0307008, and JP22uk1024002). These agencies had no further role in the study design, collection, analysis, interpretation of the data, the writing of the report, or the decision to submit the paper for publication.

## CRediT authorship contribution statement

**Hirotsugu Kawashima:** Conceptualization, Investigation, Formal analysis, Writing – original draft, Writing – review & editing. **Shimpei Yamasaki:** Conceptualization, Investigation, Formal analysis. **Manabu Kubota:** Formal analysis, Writing – review & editing. **Masaaki Hazama:** Investigation. **Yasutaka Fushimi:** Investigation. **Jun Miyata:** Writing – review & editing. **Toshiya Murai:** Formal analysis, Writing – review & editing. **Taro Suwa:** Conceptualization, Investigation, Writing – review & editing.

## Declaration of Competing Interest

The authors declare that they have no known competing financial interests or personal relationships that could have appeared to influence the work reported in this paper.
