# Supplementary material for: Commonalities and differences in ECT-induced gray matter volume change between depression and schizophrenia
Source: Neuroimage Clin. 2023 May 3;38:103429. doi: 10.1016/j.nicl.2023.103429 (PMC10193002; doi:10.1016/j.nicl.2023.103429)
Supplement: Supplementary data 1 [file mmc1.docx]

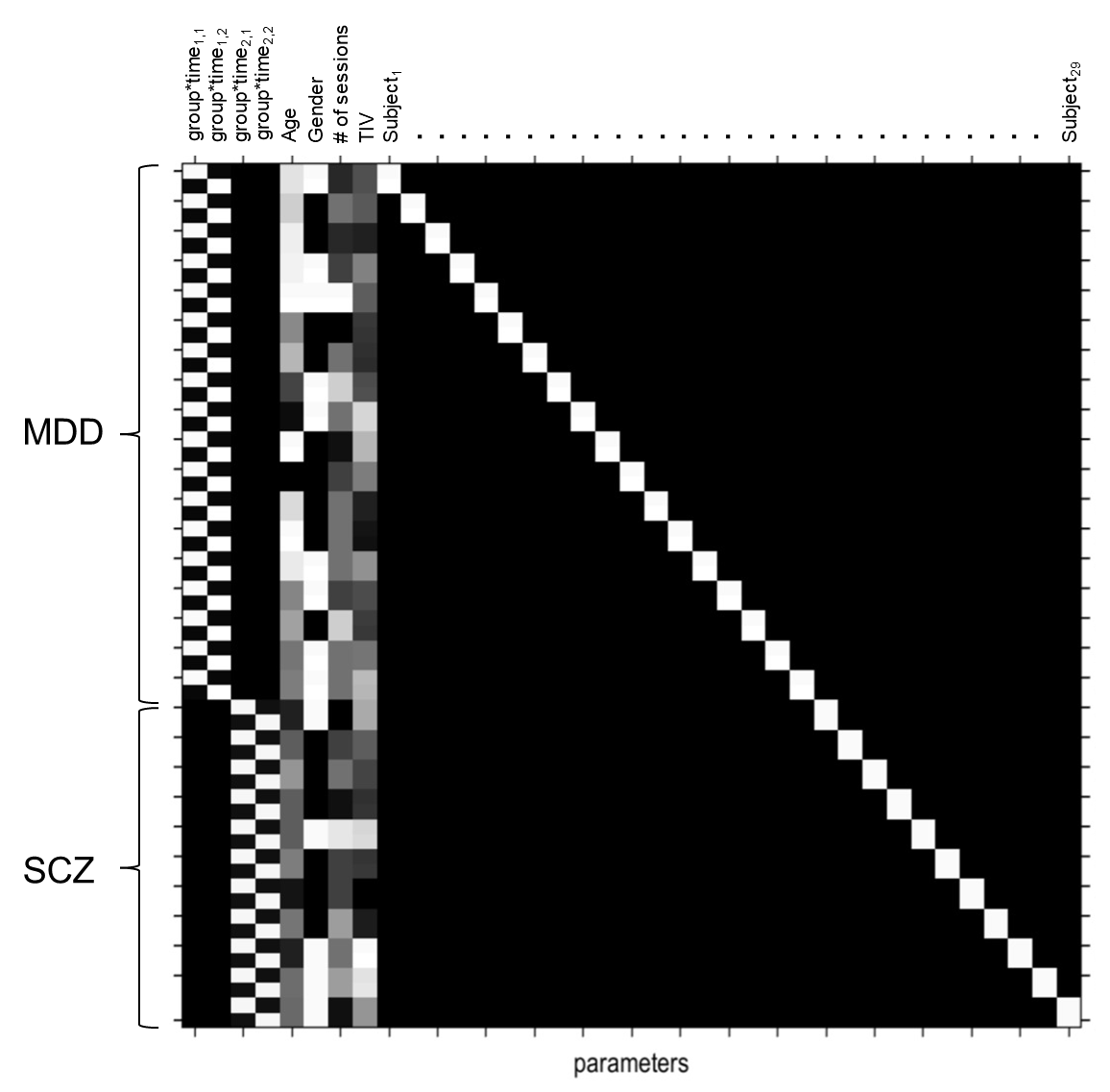


**Supplementary Fig. 1: Design matrix of the two-way repeated- measures ANCOVA in SPM12.**

MDD, Major Depressive Disorder; SCZ, Schizophrenia; # of sessions, Total number of ECT sessions; TIV, Total Intracranial Volume.


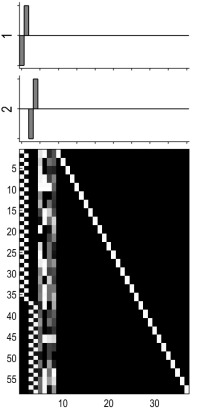

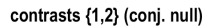


**Supplementary Fig. 2: Contrast used for the conjunction analysis in SPM12.**

The contrast 1 and 2 represent the simple main effects of time in MDD and SCZ groups respectively.

MDD, major depressive disorder; SCZ, schizophrenia.

*p=0.042

**p=0.0003

**

*


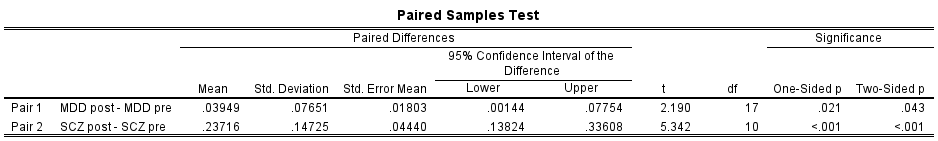


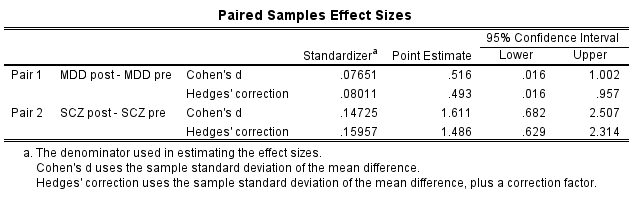


**Supplementary Fig. 3: The changes of grey matter volume (GMV) after ECT in the cluster showing significant interaction effect of group by time in the two-way repeated-measures analysis of variance.**

Paired t-tests revealed significant GMV increases after ECT in both groups, but more prominent in the SCZ group (Cohen’s d = 1.611) than in the MDD group (Cohen’s d = 0.516). Error bars represent standard error of the mean.

MDD, major depressive disorder; SCZ, schizophrenia.

**Supplementary Fig. 4: Schizophrenia-specific GMV increase in age-matched groups.**

Two-way repeated measures analysis of variance for the GMV within the cluster showing a significant interaction effect of group by time showed a prominent increase in the SCZ group. Error bars represent standard error of the mean.

GMV, gray matter volume; MDD, major depressive disorder; SCZ, schizophrenia.


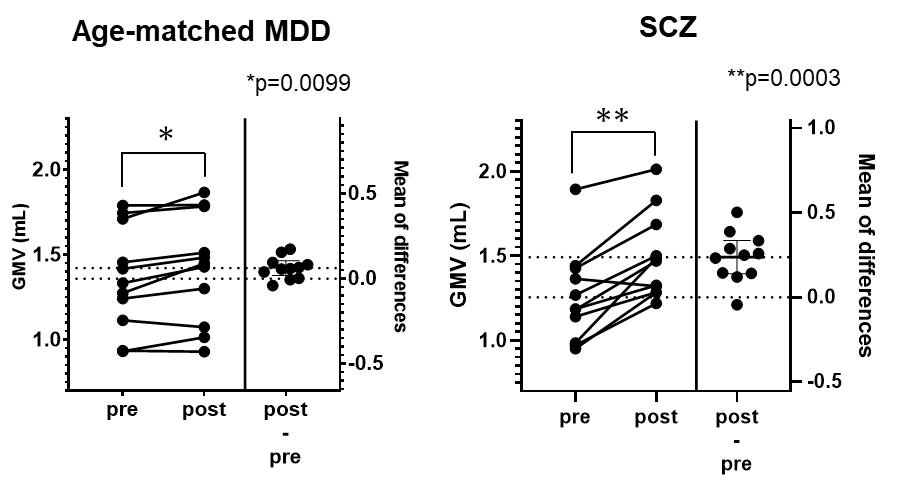


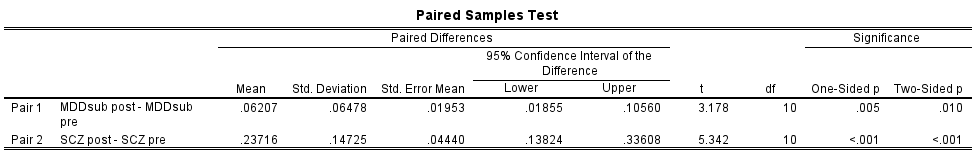


**
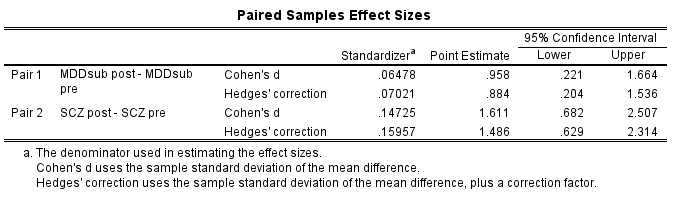
**

**Supplementary Fig. 5: Paired t-test of Gray matter volume (GMV) in age-matched groups.**

Paired t-tests revealed significant GMV increases after ECT in both groups, but more conspicuous in the SCZ group (Cohen’s d = 1.611) than in the MDD group (Cohen’s d = 0.958). Error bars represent standard error of the mean.

MDD, major depressive disorder; SCZ, schizophrenia.
