# Supplementary material for: Commonalities and differences in ECT-induced gray matter volume change between depression and schizophrenia
Source: Neuroimage Clin. 2023 May 3;38:103429. doi: 10.1016/j.nicl.2023.103429 (PMC10193002; doi:10.1016/j.nicl.2023.103429)
Supplement: Supplementary data 2 [file mmc2.pdf]

Table S1

Brain regions showing significant increase of GMV in MDD patients

No.	Brain regions	Cluster			MNI coordinate			Peak	AAL3			
		size	p(FWE-corr)	p(FDR-corr)	X	Y	Z	T value	# voxels	structure	# voxels	structure
1	Left Insula/Hippocampus/Amygdala/Temporal	3640	0.0000	0.0000	-43.5	6	4.5	7.77	795	Insula_L	359	Rolandic_Oper_L
					-18	-7.5	-15	6.67	671	Hippocampus_L	284	Temporal_Sup_L
					-34.5	12	10.5	6.30	668	Temporal_Pole_Sup_L	215	Amygdala_L
2	Right/Left MCC/SMC/Precuneus	4698	0.0000	0.0000	6	-4.5	48	7.12	1095	Supp_Motor_Area_R	610	Cingulate_Mid_R
					4.5	-13.5	48	5.91	870	Supp_Motor_Area_L	378	Precuneus_L
					-3	0	48	5.35	729	Cingulate_Mid_L	324	Precuneus_R
3	Left Temporal	3721	0.0000	0.0000	-63	-52.5	-10.5	7.10	2102	Temporal_Mid_L	43	Temporal_Sup_L
					-55.5	-33	0	6.49	519	Temporal_Inf_L	39	Occipital_Inf_L
					-51	12	-30	5.74	132	Temporal_Pole_Mid_L	29	Temporal_Pole_Sup_L
4	Left Occipital/Parietal	1277	0.0001	0.0001	-28.5	-81	31.5	6.74	569	Occipital_Mid_L	98	Parietal_Inf_L
					-24	-69	45	5.43	179	Parietal_Sup_L		
					-28.5	-87	24	5.16	106	Occipital_Sup_L		
5	Right Rolandic Operculum/Insula	1894	0.0000	0.0000	40.5	-1.5	15	6.43	783	Rolandic_Oper_R	53	Frontal_Inf_Oper_R
					49.5	-30	28.5	5.15	481	Insula_R	2	Heschl_R
					45	-18	21	5.07	131	SupraMarginal_R	2	Putamen_R
6	Right Parietal/Postcentral	796	0.0030	0.0017	63	-21	46.5	6.25	240	Parietal_Sup_R	100	Angular_R
					36	-69	52.5	4.48	181	Parietal_Inf_R	53	Occipital_Sup_R
					48	-36	58.5	4.32	107	Postcentral_R	52	SupraMarginal_R
7	Right/Left Caudate	1668	0.0000	0.0000	-4.5	7.5	3	6.16	415	Caudate_L	94	ACC_sub_L
					1.5	10.5	-4.5	5.45	234	Caudate_R	75	Olfactory_L
					7.5	7.5	15	5.27	119	Olfactory_R	45	N_Acc_L
8	Left Precentral/SFG/MFG	945	0.0011	0.0007	-27	-9	58.5	6.12	344	Precentral_L		
					-27	10.5	58.5	5.21	343	Frontal_Mid_2_L		
					-21	36	42	5.00	239	Frontal_Sup_2_L		
9	Right Hippocampus/Parahippocampal/Amygdala	1151	0.0003	0.0002	21	-12	-15	5.85	674	Hippocampus_R		
					13.5	-6	-15	5.67	124	ParaHippocampal_R		
					28.5	-16.5	-13.5	5.43	16	Amygdala_R		
10	Right STG/MTG	727	0.0050	0.0025	51	-19.5	-4.5	5.51	596	Temporal_Sup_R		
					52.5	-31.5	3	4.90	127	Temporal_Mid_R		
					45	-13.5	-1.5	3.48				
11	Left Parietal/Postcentral	626	0.0105	0.0048	-49.5	-39	55.5	5.38	420	Parietal_Inf_L		
					-40.5	-61.5	55.5	4.45	101	Postcentral_L		
					-43.5	-51	57	4.22	24	Parietal_Sup_L		
12	Right SFG/SMC	1155	0.0003	0.0002	30	22.5	58.5	5.22	863	Frontal_Sup_2_R		
					28.5	1.5	66	4.82	80	Supp_Motor_Area_R		
					28.5	31.5	54	4.75	38	Frontal_Mid_2_R		
13	Right Temporal	520	0.0241	0.0103	31.5	15	-28.5	4.89	345	Temporal_Pole_Sup_R	12	Temporal_Pole_Mid_R
					40.5	9	-21	4.85	49	Temporal_Sup_R	10	OFCpost_R
					45	1.5	-15	4.28	28	ParaHippocampal_R	5	Insula_R

Table S2

Brain regions showing significant increase of GMV in SCZ patients

No.	Brain regions	Cluster			MNI coordinate			Peak	AAL3			
		size	p(FWE-corr)	p(FDR-corr)	X	Y	Z	T value	# voxels	structure	# voxels	structure
1	Right/Left SFC/ACC/SMC	10747	0.0000	0.0000	0	46.5	9	7.00	2767	Frontal_Sup_Medial_L	676	Frontal_Sup_Medial_R
					-3	42	-1.5	6.93	923	ACC_pre_L	622	Rectus_L
					-3	3	55.5	6.54	892	Supp_Motor_Area_L	606	ACC_pre_R
2	Left Temporal/Insula/Hippocampus/Amygdala	4381	0.0000	0.0000	-28.5	6	-25.5	6.57	1397	Temporal_Pole_Sup_L	213	Frontal_Inf_Orb_2_L
					-43.5	6	4.5	6.38	1012	Insula_L	200	Amygdala_L
					-34.5	15	9	6.27	293	Temporal_Sup_L	167	ParaHippocampal_L
3	Right Temporal/Insula/Hippocampus/Amygdala	2748	0.0000	0.0000	39	9	-21	6.24	882	Temporal_Pole_Sup_R	238	Insula_R
					13.5	18	-22.5	6.20	391	Rolandic_Oper_R	137	OFCpost_R
					58.5	7.5	-1.5	5.96	249	ParaHippocampal_R	89	Temporal_Sup_R
4	Left MFC/SFC	639	0.0096	0.0095	-25.5	27	43.5	5.49	337	Frontal_Mid_2_L		
					-21	36	40.5	4.91	302	Frontal_Sup_2_L		
					-18	49.5	37.5	4.52				

Table S3

Common Brain regions showing significant increase of GMV in MDD and SCZ patients

No.	Brain regions	Cluster			MNI coordinate			Peak	AAL3			
		size	p(FWE-corr)	p(FDR-corr)	X	Y	Z	T value	# voxels	structure	# voxels	structure
1	Left Temporal/Insula/Hippocampus/Amygdala	2086	0.0000	0.0000	-43.5	6	4.5	6.38	651	Temporal_Pole_Sup_L	167	Amygdala_L
					-43.5	6	-19.5	5.97	622	Insula_L	114	Hippocampus_L
					-33	13.5	10.5	5.95	181	Temporal_Sup_L	80	Rolandic_Oper_L
2	Right/Left Caudate/Olfactory	946	0.0011	0.0010	0	10.5	-4.5	5.42	294	Caudate_L	65	Olfactory_L
					-3	10.5	4.5	5.32	92	Olfactory_R	59	ACC_sub_L
					-9	9	15	4.95	65	Caudate_R	22	Frontal_Med_Orb_R
3	Right/Left SMC/MCC/SFC	1273	0.0001	0.0002	-1.5	6	52.5	5.08	583	Supp_Motor_Area_L	125	Cingulate_Mid_L
					0	18	48	4.49	284	Supp_Motor_Area_R	98	Cingulate_Mid_R
					-1.5	25.5	40.5	4.38	165	Frontal_Sup_Medial_L	16	Frontal_Sup_Medial_R
4	Right Temporal/ParaHippocampal	494	0.0298	0.0202	31.5	15	-28.5	4.89	337	Temporal_Pole_Sup_R	10	OFCpost_R
					40.5	9	-21	4.85	34	Temporal_Sup_R	10	Temporal_Pole_Mid_R
					45	1.5	-15	4.28	28	ParaHippocampal_R	5	Insula_R

Table S4
Demographic and clinical characteristics used in post-hoc age-matched subset analysis.

Demographic and clinical variables		Age-matched MDD patients (n = 11)		SCZ patients (n = 11)		P-value
Mean age, years (SD)		52.1	(14.7)	44.9	(7.9)	0.173 §
Sex, female (*n* [%])		5	(45.5)	5	(45.5)	1.000‡
Psychotic Depression (*n* [%])		4	(36.4)			
Catatonia (*n* [%])		1	(9.1)	9	(81.8)	
Mean number of ECT sessions (S.D.)		9.9	(2.5)	9.1	(2.9)	0.502 §
Clinical Score						
mean HDRS (S.D.)	pre ECT	27.2	(7.8)			<0.001¶
	post ECT	10.1	(7.2)			
mean BPRS (S.D.)	pre ECT			57.3	(9.8)	<0.001¶
	post ECT			18.3	(9.2)	

MDD: major depressive disorder, SCZ: schizophrenia, SD: standard deviation, HDRS: Hamilton Depression Rating Scale, BPRS: Brief Psychotic Rating Scale

§ : two-sample t-test, ‡: Chi-squared test, ¶: paired t-test
